# Urinary Taurine Excretion and Risk of Late Graft Failure in Renal Transplant Recipients

**DOI:** 10.3390/nu11092212

**Published:** 2019-09-13

**Authors:** Adrian Post, M. Yusof Said, Antonio W. Gomes-Neto, Jennifer van der Krogt, Pim de Blaauw, Stefan P. Berger, Johanna M. Geleijnse, Karin Borgonjen, Else van den Berg, Harry van Goor, Gerald Rimbach, Ido P. Kema, Dimitrios Tsikas, M. Rebecca Heiner-Fokkema, Stephan J. L. Bakker

**Affiliations:** 1 Department of Internal Medicine, University Medical Center Groningen, University of Groningen, 9713 GZ Groningen, The Netherlands; m.y.said@umcg.nl (M.Y.S.); a.w.gomes.neto@umcg.nl (A.W.G.-N.); s.p.berger@umcg.nl (S.P.B.); e.van.den.berg@umcg.nl (E.v.d.B.); s.j.l.bakker@umcg.nl (S.J.L.B.); 2 Department of Laboratory Medicine, University Medical Center Groningen, University of Groningen, 9713 GZ Groningen, The Netherlands; j.van.der.krogt@umcg.nl (J.v.d.K.); p.de.blaauw@umcg.nl (P.d.B.); i.p.kema@umcg.nl (I.P.K.); m.r.heiner@umcg.nl (M.R.H.-F.); 3 Department of Human Nutrition and Health, Wageningen University, 6708 PB Wageningen, The Netherlands; marianne.geleijnse@wur.nl (J.M.G.); karin.borgonjen@wur.nl (K.B.); 4 Department of Pathology and Medical Biology, University Medical Center Groningen, University of Groningen, 9713 GZ Groningen, The Netherlands; h.van.goor@umcg.nl; 5 Institute of Human Nutrition and Food Science, Christian-Albrechts-University of Kiel, 24118 Kiel, Germany; rimbach@foodsci.uni-kiel.de; 6 Institute of Toxicology, Core Unit Proteomics, Hannover Medical School, Carl-Neuberg-Str. 1, 30625 Hannover, Germany; tsikas.dimitros@mh-hannover.de

**Keywords:** renal transplant recipients, taurine, taurine excretion, graft survival

## Abstract

Taurine is a sulfur containing nutrient that has been shown to protect against oxidative stress, which has been implicated in the pathophysiology leading to late graft failure after renal transplantation. We prospectively investigated whether high urinary taurine excretion, reflecting high taurine intake, is associated with low risk for development of late graft failure in renal transplant recipients (RTR). Urinary taurine excretion was measured in a longitudinal cohort of 678 stable RTR. Prospective associations were assessed using Cox regression analyses. Graft failure was defined as the start of dialysis or re-transplantation. In RTR (58% male, 53 ± 13 years old, estimated glomerular filtration rate (eGFR) 45 ± 19 mL/min/1.73 m^2^), urinary taurine excretion (533 (210–946) µmol/24 h) was significantly associated with serum free sulfhydryl groups (β = 0.126; *P* = 0.001). During median follow-up for 5.3 (4.5–6.0) years, 83 (12%) patients developed graft failure. In Cox regression analyses, urinary taurine excretion was inversely associated with graft failure (hazard ratio: 0.74 (0.67–0.82); *P* < 0.001). This association remained significant independent of potential confounders. High urinary taurine excretion is associated with low risk of late graft failure in RTR. Therefore, increasing taurine intake may potentially support graft survival in RTR. Further studies are warranted to determine the underlying mechanisms and the potential of taurine supplementation.

## 1. Introduction

Globally, renal transplantation becomes more prevalent each year [1], and renal transplantation is the preferred treatment for end-stage renal disease. However, donor kidneys remain sparse and patients often spend years on waiting lists or depend on a sacrifice by a family member or friend in the form of a living donation. Therefore, protection of donated kidneys and improving long-term graft survival is a major clinical, as well as ethical, necessity. Due to improvements in surgical techniques, immunosuppressant drugs and postoperative care, the one-year survival rate has steadily improved, but late graft failure occurring beyond the first year after transplantation has not decreased substantially in the last decades [2]. Though the exact mechanisms leading to late graft failure are not fully understood, oxidative stress has been implicated [3,4,5,6].

Taurine is an amino sulfonic acid found in high concentrations in many cells, where it is implicated in numerous physiological functions. In some species, e.g., cats, taurine is an essential nutrient, but in humans, it is considered a conditionally essential nutrient [7,8,9]. While taurine can be synthesized endogenously, humans primarily depend on their diet for taurine, where it is mostly found in seafood and meat [10,11]. Although taurine fulfills a wide array of function, its cytoprotective actions have received the most attention [7,12,13,14]. Taurine has been demonstrated to have renoprotective effects in several animal studies [15,16,17,18]. In rats, preconditioning of donor organs with taurine protected grafts during transplantation against both cold ischemia and ischemia reperfusion injury [19]. However, the effects of taurine on long term graft survival in humans are not known. Based on the acknowledged cytoprotective properties of taurine, we hypothesize that a higher taurine exposure is associated with improved renal graft survival. Therefore, the primary aim of our study was to investigate whether 24 h urinary taurine excretion is associated with graft failure in stable outpatient renal transplant recipients.

## 2. Materials and Methods

### 2.1. Study Population

This observational prospective study was conducted in a large single center renal transplant recipients (RTR) cohort, as previously described [20,21]. In short, all adult (≥18 years old) RTR without known or apparent systemic illnesses (i.e., malignancies, opportunistic infections) who visited the outpatient clinic of the University Medical Center Groningen between November 2008 and June 2011 were invited to participate in this prospective cohort study. RTR were all transplanted at the University Medical Center Groningen and had no history of drug or alcohol addiction. Of the 817 initially invited RTR, 706 (87%) signed written informed consent to participate in this study. We excluded subjects with missing data on urinary taurine excretion, i.e., 28 cases, from the statistical analyses, which resulted in 678 cases eligible for analyses. To compare urinary taurine excretion of RTR with patients without renal disease, we included 275 healthy kidney donors of whom biomaterial was collected before kidney donation. The study protocol was approved by the University Medical Center Groningen institutional ethical review board (Medical ethical committee 2008/186) and adhered to the Declarations of Helsinki.

### 2.2. Clinical Parameters

All measurements were performed during a morning visit to the outpatient clinic after an 8- to 12-h overnight fasting period. Blood pressure was measured (in millimeters of mercury) with a semiautomatic device (Dinamap 1846; Critikon, Tampa, FL, USA) according to a strict protocol as previously described [22]. Information on participants’ health status, medical history, and medication use was obtained from patient records. Information on smoking behavior and alcohol intake was obtained by using a questionnaire. Participants were classified as current, former, or never smokers. Alcohol intake was split into no intake, low intake (0–5 g/24 h in females and 0–10 g/24 h in males) and high intake (>5 g/24 h in females and >10 g/24 h in males). Body weight and height were measured with participants wearing indoor clothing without shoes. Body mass index (BMI) was calculated as weight in kilograms divided by height in meters squared and body surface area (BSA) was calculated using the formula of Du Bois and Du Bois [23]. Diabetes mellitus was diagnosed according to American Diabetes Association criteria (2017) as having a fasting plasma glucose concentration ≥7.0 mmol/L or the use of an antidiabetic medication [24,25]. Hypertension was defined as predialysis systolic blood pressure >140 mmHg and/or diastolic blood pressure >90 mmHg or use of antihypertensive drugs.

### 2.3. Dietary Intake

Information on dietary intake was obtained from a validated semiquantitative food-frequency questionnaire (FFQ), which was linked to the Dutch food composition table (NEVO) to compute the intake of energy, macronutrients and micronutrients [26]. It was not possible to compute intake of taurine using the FFQ, because the Dutch food composition table does not contain data on taurine contents of food items. Because not all participants completed or returned the FFQ, 641 RTR and 183 controls had data available on dietary intake. The FFQ inquired about intakes of 177 food items during the past month with seasonal variations taken into account. For each item, the frequency was recorded in times per day, week, or month. The number of servings was expressed in natural units (e.g., slice of bread or apple) or household measures (e.g., cup or spoon). The questionnaire was self-administered and filled out at home. Every FFQ was checked for completeness by a trained researcher, and inconsistent answers were verified with the patients. Validation of the FFQ in RTR was assessed, as previously reported [27]. Dietary data were converted into daily nutrient intakes with the use of the Dutch Food Composition Table of 2006 [28]. Dietary intakes were adjusted for total energy intake (kcal/24 h) according to the residual method [29].

### 2.4. Laboratory Measurements

All participants were instructed to collect a 24-h urine sample according to a strict protocol at the day before their visit to the outpatient clinic. Urine was collected under oil and chlorhexidine was added as an antiseptic agent. Urine taurine concentrations were analyzed by ultra-high performance liquid chromatography triple quadrupole mass spectrometry analysis (UHPLC-MS/MS). In short, samples were derivatized using AccQ•Tag derivatization reagent according to the manufacturer’s protocol (Waters Corporation, Milford, MA, USA) and separated using a Cortecs UPLC C18 (1.6 µm pore size, 150 × 2.1 mm) analytical column. Taurine was detected using positive-ion electrospray ionization in multiple reaction monitoring mode, using the following transitions: m/z 295.9 -> 171.0 for taurine and 335 -> 171.0 for the internal standard (^13^C_6_, ^15^N_3_-histidine). Data were analyzed using MultiQuant MD 3.0.2 (Sciex). Inter-assay precision was monitored using three urine pool samples. The inter-assay precisions were 8.5% at 270.4 µmol/L, 9.8% at 594.7 µmol/L and 7.9% at 762.0 µmol/L. The upper limit of detection was 1200 µmol/L and values above 1200 µmol/L were reported as 1201 µmol/L. Urinary protein concentration was determined by means of the Biuret reaction (MEGA AU 510; Merck Diagnostica, Darmstadt, Germany). Proteinuria was defined as urinary protein excretion ≥0.5 g/24 h. Upon completion of the 24-h urine collection, fasting venous blood samples anti-coagulated with lithium-heparin, sodium-fluoride and potassium-ethylenediaminetetraacetic acid (EDTA) were obtained the following morning. For routine clinical chemistry assays, heparin plasma was analyzed spectrophotometrically on the same morning using automated and validated routine methods (Roche Diagnostics, Basel, Switzerland). Fasting glucose was assessed from fluoride plasma on the same morning on the same equipment. EDTA plasma was separated in small aliquots and stored frozen at −80°C for later use. Aliquots of the 24 h urine collection were also stored frozen at −80°C for later use. Free sulfhydryl groups in serum were quantified using Ellman’s reagent. Human leukocyte antigen I (HLA-I) and HLA-II antibodies were quantified using an ELISA (LATM205, One Lambda, Canoga Park, CA, USA). Urinary inorganic sulfate was measured with a validated ion-chromatography method (type 861; Metrohm, Herisau, Switzerland). Urinary thiosulfate was determined using a validated HPLC method [20]. Renal function was assessed by the estimated glomerular filtration rate (eGFR) based on the Chronic Kidney Disease Epidemiology Collaboration Creatinine Cystatin C (CKD-EPI-sCr-CysC) equation [30].

### 2.5. Graft Failure

The endpoint of this study was graft failure. Graft failure was defined as re-transplantation or return to dialysis and was censored for death. The continuous surveillance system of the outpatient program ensured that there was up-to-date information on patient status. Endpoints were recorded until September 2015 by a qualified physician, with no loss to follow-up.

### 2.6. Statistical Analysis

Data analyses and computations were performed with SPSS 24.0 software (IBM, Armonk, NY, USA), Stata SE version 15 (StataCorp, College Station, TX, USA), R version 3.5.1 software (The R-Foundation for Statistical Computing), and GraphPad Prism version 5 (GraphPad Software). Baseline data are presented as means ± standard deviation for normally distributed data, as medians (interquartile range) for non-normally distributed data, and as numbers (percentages) for nominal data. A two-sided *P* < 0.05 was considered to indicate statistical significance. Differences between RTR and healthy controls were tested with a t-test for independent samples, the Mann–Whitney U test, or the chi-squared test. Cross-sectional associations of urinary taurine excretion with baseline variables were studied using linear regression models. Regression coefficients were given as standardized beta values, the latter referring to the number of standard deviations a dependent variable changes per standard deviation increase of the independent variable, thereby allowing for comparison of the strength of the associations of different variables. Cox regression analyses were employed to investigate the association of urinary taurine excretion, with graft failure. Secondarily, analyses were also performed for urinary taurine concentration and urinary taurine/creatinine ratio. Cox regression models were built in a stepwise fashion to avoid overfitting and to keep the number of predictors in proportion to the number of events [31]. Adjustments were made for a priori selected variables and for potentially relevant variables identified using linear regression analyses. A priori selected variables were basic potential confounders (model 2), cardiovascular risk factors (model 3) and transplantation related factors (model 4). Basic potential confounders were defined as age, sex, weight, height, eGFR and proteinuria. Cardiovascular risk factors were defined as total cholesterol, High-density lipoprotein (HDL) cholesterol, triglycerides, systolic blood pressure, antihypertensive treatment, smoking (current, ex, or never), presence of diabetes, medical history of coronary intervention, myocardial infarction, cerebrovascular accident (CVA) and/or transient ischemic attack (TIA). Transplantation related factors were defined as donor type, total dialysis time, time from transplantation and baseline, cold ischemia time, calcineurin inhibitor (CNI) usage, proliferation inhibitor usage, and the number of transplantations up to baseline. Potentially relevant variables were selected if the *P* value for the association with urinary taurine excretion (Table 1) was <0.05. In model 5, we adjusted for potentially relevant variables that have not been adjusted for in previous models. Schoenfeld residuals of urinary taurine excretion, urinary taurine concentration and urinary taurine/creatinine ratio were checked in R, the assumption of proportional hazards was not violated for urinary taurine excretion, urinary taurine concentration and the urinary taurine/creatinine ratio (*P* = 0.77, *P* = 0.65 and *P* = 0.55, respectively). Potential interactions for the covariates age, sex, body mass index (BMI), hypertension, diabetes, renal function, proteinuria, smoking status, alcohol intake and time between baseline and transplantation were assessed by calculating interaction terms. To determine the optimal cut off value (Youden index) of urinary taurine excretion for prediction of graft failure in RTR, the ‘survivalROC’ package in R was used. To visualize the continuous associations of urinary taurine excretion, urinary taurine concentration and urinary taurine/creatinine ratio with graft failure, log_2_-transformed urinary taurine excretion, urinary taurine concentration and urinary taurine/creatinine ratio, as continuous variables, were individually plotted against the risk of graft failure.

## 3. Results

### 3.1. Baseline Characteristics of RTR and Controls

Characteristics of RTR versus controls are shown in supplementary Table S1. For RTR, the median time between transplantation and baseline measurements was 5.3 (1.8–11.5) years. Urinary taurine excretion (533 (210–946) µmol/24 h versus 477 (253–943) µmol/24 h; *P* = 0.92), urinary taurine concentration (216 (87–415) µmol/L versus 199 (100–394) µmol/L; *P* = 0.85) and urinary taurine/creatinine ratio (46 (20–80) µmol/mmol versus 41 (21–66) µmol/mmol; *P* = 0.21) were similar in RTR and controls, respectively. The two groups were also similar with respect to age, height and BSA, though RTR had a higher BMI (26.6 ± 4.8 kg/m^2^ versus 26.0 ± 3.5 kg/m^2^; *P* = 0.02). Men were overrepresented in the RTR group compared with the control group (58% male versus 47% male; *P* = 0.002). As anticipated, eGFR was significantly lower in RTR than in controls (45 ± 19 mL/min/1.73 m^2^ versus 92 ± 16 mL/min/1.73 m^2^; *P* < 0.001). Animal-based protein intake was similar in RTR and controls (51 ± 13 versus 51 ± 12 g/24 h; *P* = 0.98), while energy intake (2172 ± 619 versus 2294 ± 730 kcal/24 h; *P* = 0.02), total protein intake (82 ± 12 g/24 h versus 85 ± 15 g/24 h; *P* = 0.007), plant protein intake (31 ± 6 g/24 h versus 33 ± 8 g/24 h; *P* < 0.001), cysteine intake (1187 ± 172 mg/24 h versus 1238 ± 190 mg/24 h; *P* = 0.001), total fat intake (88 ± 16 g/24 h versus 94 ± 20 g/24 h; *P* = 0.001) and total carbohydrate intake (249 ± 63 g/24 h versus 258 ± 49 g/24 h; *P* = 0.03) were lower in RTR compared to controls.

### 3.2. Linear Regression

Selected baseline characteristics of RTR and linear regression analysis are shown in Table 1. Univariate, urinary taurine excretion was positively associated with serum free sulfhydryl groups (β = 0.249; *P* < 0.001), urinary taurine concentration (β = 0.962; *P* < 0.001), urinary taurine/creatinine ratio (β = 0.969; *P* < 0.001), male sex (β = 0.374; *P* < 0.001), never smoking status (β = 0.083; *P* = 0.04), weight (β = 0.121; *P* = 0.002), height (β = 0.266; *P* < 0.001), BSA (β = 0.196; *P* < 0.001), use of proliferation inhibitors (β = 0.095; *P* = 0.01), prednisolone dosage (β = 0.135; *P* < 0.001), eGFR (β = 0.252; *P* < 0.001), creatinine clearance (β = 0.361; *P* < 0.001) and urinary excretion of sodium (β = 0.288; *P* < 0.001), chloride (β = 0.268; *P* < 0.001), sulfate (β = 0.324; *P* < 0.001), thiosulfate (β = 0.285; *P* < 0.001) and creatinine (β = 0.397; *P* < 0.001). In contrast, urinary taurine excretion was inversely associated with antihypertensive drugs (β = −0.082; *P* = 0.03), N-terminal pro b-type natriuretic peptide (NT-proBNP) (β = −0.196; *P* < 0.001), total cholesterol (β = −0.174; *P* < 0.001), HDL cholesterol (β = −0.112; *P* = 0.004), LDL cholesterol (β = −0.094; *P* = 0.02), triglycerides (β = −0.168; *P* < 0.001), use of statins (β = −0.080; *P* = 0.04), use of antidiabetic drugs (β = −0.093; *P* = 0.02), time between baseline and transplantation (β = −0.169; *P* < 0.001), HLA-I antibodies (β = −0.110; *P* = 0.004), HLA-II antibodies (β = −0.133; *P* = 0.001), serum creatinine (β = −0.129; *P* < 0.001) and proteinuria (β = −0.107; *P* = 0.005). Adjustment for age, sex and eGFR revealed an association of urinary taurine excretion with past smoking status (β = −0.087; *P* = 0.02), polycystic kidney disease (β = 0.081; *P* = 0.02) and use of calcineurin inhibitors (β = 0.082; *P* = 0.02). In addition, adjustment for age, sex and eGFR strengthened the associations of urinary taurine excretion with urinary taurine/creatinine ratio (β = 0.993; *P* < 0.001), never smoking status (β = 0.113; *P* = 0.002) and time between baseline and transplantation (β = −0.180; *P* < 0.001). In contrast, adjustment for age, sex and eGFR somewhat weakened the associations of urinary taurine excretion with serum free sulfhydryl groups (β = 0.126; *P* = 0.001), total cholesterol (β = −0.120; *P* < 0.001), triglycerides (β = −0.118; *P* = 0.001), use of antidiabetic drugs (β = −0.079; *P* = 0.03), eGFR (β = 244; *P* < 0.001), creatinine clearance (β = 0.314; *P* < 0.001), proteinuria (β = −0.086; *P* = 0.02), and urinary excretion of sodium (β = 0.179; *P* < 0.001), chloride (β = 0.151; *P* < 0.001), sulfate (β = 0.199; *P* < 0.001), thiosulfate (β = 0.170; *P* < 0.001) and creatinine (β = 0.265; *P* < 0.001). The associations of urinary taurine excretion with weight, height, BSA, use of antihypertensive drugs, NT-proBNP, HDL cholesterol, LDL cholesterol, use of statins, use of proliferation inhibitors, prednisolone dosage, HLA-I antibodies, HLA-II antibodies and serum creatinine all disappeared after adjustment for age, sex and eGFR (all *P* > 0.05).

### 3.3. Dietary Intake

Univariate, urinary taurine excretion associated positively with the intake of energy (β = 0.126; *P* = 0.002), total protein (β = 0.190; *P* < 0.001), animal protein (β = 0.133; *P* = 0.001), methionine (β = 0.176; *P* < 0.001), cysteine (β = 0.238; *P* < 0.001), meat (β = 0.223; *P* < 0.001), fish (β = 0.104; *P* = 0.01), plant protein (β = 0.110; *P* = 0.006), total fat (β = 0.212; *P* < 0.001) and total carbohydrate (β = 0.186; *P* < 0.001). In contrast, urinary taurine was inversely associated with fruit intake (β = −0.117; *P* = 0.004). After adjustment for age, sex and eGFR the associations of urinary taurine excretion with the intake of energy, total protein, cysteine, plant protein, fruit, total fat and total carbohydrate disappeared (All > 0.05), while the associations with animal protein intake (β = 0.085; *P* = 0.03), methionine intake (β = 0.077; *P* = 0.05), meat intake (β = 0.151; *P* < 0.001) and fish intake (β = 0.096; *P* = 0.008) remained.

### 3.4. Graft Failure

**Figure 1:**
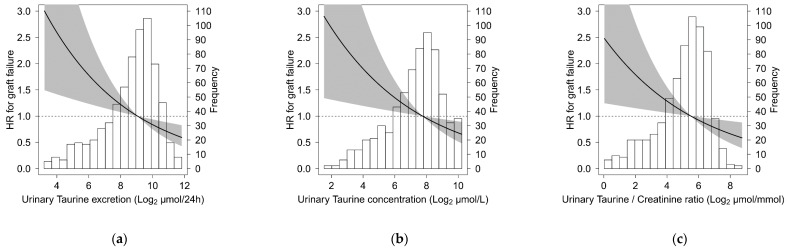
Continuous association of urinary taurine excretion (**a**), urinary taurine concentration (**b**) and urinary taurine/creatinine ratio (**c**) with death-censored graft failure. Urinary taurine excretion, urinary taurine concentration and urinary taurine/creatinine ratio were log_2_-transformed for prospective analysis. The histograms depict the distribution of urinary taurine excretion, urinary taurine concentration and urinary taurine/creatinine ratio. The black line shows the adjusted hazard ratio (HR) and the gray areas correspond to the 95% confidence interval (CI). The adjusted association was adjusted for age, sex, weight, height, eGFR and proteinuria. *P* values were 0.002, 0.007 and 0.01, respectively.

During median follow-up for 5.3 (4.5–6.0) years, 83 (12%) patients developed graft failure. Patients experiencing graft failure had almost two-fold lower urinary taurine excretion (320 (57–676) µmol/24 h versus 553 (239–964) µmol/24 h; *P* < 0.001), urinary taurine concentration (105 (30–290) µmol/L versus 225 (101–430) µmol/L; *P* = 0.001) and urinary taurine/creatinine ratio (26 (7–58) µmol/mmol versus 48 (22–82) µmol/mmol; *P* < 0.001) compared to patients not experiencing graft failure during follow-up. Prospective analyses of the association between urinary taurine excretion, urinary taurine concentration and urinary taurine/creatinine ratio with graft failure in RTR are shown in Table 2. In Cox regression analyses, log_2_ transformed urinary taurine excretion (HR: 0.74 (0.67–0.082); *P* < 0.001), urinary taurine concentration (HR: 0.75 (0.67–0.84); *P* < 0.001) and urinary taurine/creatinine ratio (HR: 0.74 (0.66–0.83); *P* < 0.001) were inversely associated with graft failure. After adjustment for relevant covariates (age, sex, weight, height, eGFR and proteinuria) the associations of urinary taurine excretion (HR: 0.83 (0.74–0.93); *P* = 0.002), urinary taurine concentration (HR: 0.84 (0.74–0.95); *P* = 0.007) and urinary taurine/creatinine ratio (0.85 (0.75–0.96); *P* = 0.01) were somewhat weakened, but remained significant. Further adjustment for cardiovascular risk factors (model 3), transplantation related factors (model 4), polycystic kidney disease and urinary excretion of sodium, chloride, sulfate, thiosulfate and creatinine (model 5) did not materially change the associations. No significant interactions terms were found for the selected covariates (All *P* > 0.05). Analyses using creatinine clearance instead of eGFR yielded similar results (Table S2). The optimal cutoff value (Youden index) of urinary taurine excretion for prediction of graft failure was 285.4 µmol/24 h. At this cut off value, there was a sensitivity of 57% and a specificity of 75% for prediction of graft failure. The associations of log_2_ transformed urinary taurine excretion, urinary taurine concentration and urinary taurine/creatinine ratio as continuous variables with death-censored graft failure are visualized in Figure 1.

## 4. Discussion

To the best of our knowledge, this is the first study that assessed urinary taurine excretion in stable, outpatient RTR and investigated the prospective association of urinary taurine excretion with late graft failure. Urinary taurine excretion did not differ between RTR and healthy controls and in RTR urinary taurine excretion was positively associated with free serum sulfhydryl groups, but not with high-sensitivity C-reactive protein. We found that high urinary taurine excretion associated with lower risk of late graft failure in RTR, independent of potential confounders.

Despite the great impact of modern immunosuppression and anti-infection prophylaxis on reducing acute graft failure, there has been little impact on long term graft survival [32]. A prelude to most late graft failures is chronic renal dysfunction, which is believed to be a combination of both immunological factors and non-immunological risk factors [33,34]. One of the non-immunological risk factors thought to contribute to chronic renal dysfunction is oxidative stress [3,35,36,37,38,39,40], representing damage to cells and tissues caused by reactive oxygen species (ROS). Compared to the general population, biomarkers of oxidative stress are increased in renal transplant recipients [40] and are even further increased in RTR with chronic transplant dysfunction [5]. In addition, in RTR, lower levels of biomarkers of oxidative stress are associated with better outcome over time [4,39]. Possible sources of these ROS in RTR could be inflammation [38,41,42], immunosuppressive drugs, e.g., ciclosporin [43,44], and renal tissue hypoxia [3,45].

Taurine is a sulfonated β-amino acid, being present in very high concentrations in most mammalian cells, comprising nearly 0.1% of the total body weight in humans [9,46]. Although taurine has been shown to influence many physiological functions, its cytoprotective effects have gained the most attention [7]. In humans, elevated taurine consumption is associated with decreased risk of hypertension and hypercholesterolemia [47,48], reduced body mass index [49], and a reduction of inflammation markers [50]. In our study, we did find a strong inverse association of urinary taurine excretion with cholesterol and triglycerides, but no associations with blood pressure, body mass index or high-sensitivity C-reactive protein. A possible reason that no association with blood pressure was found in this study could be the high frequency of use of antihypertensive drugs in RTR. Median urinary taurine excretion did not differ between RTR and healthy controls. Both the median urinary taurine in RTR of 533 µmol/24 h and in healthy controls of 477 µmol/24 h were well in line with the median urinary excretions reported for European, North American and Oceanic Caucasian participants of the World Health Organization (WHO) Cardiovascular Diseases and Alimentary Comparison (CARDIAC) study [51].

The mechanisms for the cytoprotective effects of taurine are not completely understood, but one of the primary mechanisms appears to be its antioxidative properties. Several studies investigated the scavenging activity of taurine in vitro and the results varied greatly. Concentrations ranging from 1 to 20 mM exhibited little to no scavenging activity against H_2_O_2_, superoxide anion radicals (O_2_^•−^), or hydroxyl radicals (OH^•^) [52,53], while concentrations above 20 mM exhibited a concentration dependent scavenging activity against peroxyl radicals (ROO^•^), nitric oxide (NO^•^), superoxide anion radicals (O2^•−^) and peroxynitrite (ONOO^−^), with the greatest activity being achieved at a concentration of 60 mM [54]. In vivo, extracellular taurine concentrations ranged from 10 to 100 µM and intracellular concentrations ranged from 5 to 50 mM, with the highest intracellular concentrations found in neutrophils and the retina [55].

A more universally accepted mechanism is that taurine diminishes superoxide in mitochondria by conjugating with uridine of tRNA^Leu(UUR)^, which is supported by findings that promotors of mitochondrial stress, such as ozone, bleomycin and catecholamines, respond favorably to taurine supplementation [56,57]. Another mechanism is the attenuation of the toxicity of hypochlorous acid (HOCl) produced by the myeloperoxidase (MPO) system in neutrophils. Taurine readily reacts with hypochlorous acid to produce the more stable oxidant taurine chloramine [58,59,60]. Not only is taurine chloramine less toxic, it also inhibits the production of inflammatory mediators, such as superoxide anion, nitric oxide, tumor necrosis factor-α and interleukins 6, 8 and 12 [59,60,61]. Furthermore, in macrophages, taurine chloramine increases the expressions of several antioxidant proteins, such as peroxiredoxin, thioredoxin, heme oxygenase 1 and glutathione peroxidase, partly via a Nrf2-dependent signal transduction pathway [59,60,61]. Since neutrophils play an important role in both chronic rejection and the resolution of inflammation [62], the mechanisms underlying the inverse association of urinary taurine excretion and graft failure we observed may be a combination of both antioxidation and anti-inflammatory effects. In our study, urinary taurine excretion was positively associated with free sulfhydryl groups, indicating that patients with a higher urinary taurine excretion may have a more favorable redox status [63,64,65,66,67]. Interestingly, we also found a strong association between urinary taurine excretion and creatinine excretion. It is well known that 24 h urinary excretion of creatinine is a reflection of muscle mass [68,69]. It has previously been suggested that taurine is a promising nutritional agent to counteract the effects and development and sarcopenia [70]. While we cannot determine a causal relation between urinary taurine excretion and creatinine excretion, it is clear that this association is not mediated through a higher caloric intake, as there is no association of urinary taurine excretion with energy intake. Adjustment for creatinine excretion in Cox-regression models did not materially change the association of urinary taurine excretion with graft failure.

Though the antioxidative and anti-inflammatory effects of taurine have been extensively studied, there are many other physiological functions that taurine fulfills. Taurine has been implicated in energy metabolism, neuromodulation, Ca^2+^ homeostasis, attenuation of endoplasmic reticular stress, osmoregulation [7], bile acid conjugation and maintenance of euglycemia [55]. Due to its wide variety of effects, it is possible that some of the above-mentioned functions also play a role in the found association with graft failure. In the Cox regression models, we adjusted for potential confounders, including age, sex, body composition, renal function and proteinuria. The adjustments only caused slight weakening of the association of urinary taurine excretion with graft failure, leaving a significant independent association of urinary taurine excretion with graft failure. Further adjustment for cardiovascular risk factors, transplantation related factors and urinary excretions did not materially affect the association. Analysis of the optimal cut off value for urinary taurine excretion in predicting graft failure shows that urinary taurine excretion does not perform very well if it would have been evaluated as a potential early diagnostic tool for prediction of graft failure in RTR. The aim of our study was, however, to investigate whether urinary taurine excretion might be a potential modifiable risk factor for the development of graft failure in RTR. Our finding of a significant independent association of urinary taurine excretion with graft failure suggests that this might indeed be the case.

In steady state, 24 h urinary excretion of taurine reflects a combination of dietary taurine intake and endogenous taurine synthesis from cysteine. In the latter case, cysteine is first oxidized to cysteine sulfinic acid, after which it can be further metabolized by two routes. Most cysteine sulfinic acid follows the transamination route leading to the end-product sulfate. A smaller percentage is metabolized to hypotaurine and eventually taurine. In humans, this endogenous synthesis of taurine is very limited due to low activity of the rate limiting enzyme, cysteine sulfinic acid decarboxylase [9,11,55]. Consequently, dietary taurine uptake is the major supply of taurine. Important sources for taurine in the human diet are seafood, poultry, beef and pork, processed meats and to a lesser degree dairy [11]. In contrast, plant-based foods contain little to no taurine [11]. Our data is consistent with this, as urinary taurine excretion was associated with animal protein intake, meat intake and fish intake, but not with fruit and vegetable intake. The association with animal protein intake may also underlie strong associations of urinary taurine excretion with sulfate and thiosulfate excretion, which are both end-products of the metabolism of sulfur containing amino acids [20].

After absorption, taurine is widely distributed among tissues, with the high concentrations found in the retina, brain, heart, liver, kidney and muscles [55]. Plasma taurine is subsequently conjugated with bile acids via its amine group or excreted unchanged in urine [9]. By varying taurine reabsorption, the kidneys are the primary regulators of taurine homeostasis [9,71]. In humans, a study found that 95% of an intravenous injection of [^35^S] taurine was recovered in urine; about 70% in the form of taurine and 25% in the form of sulfate [72]. After conjugation with bile acids and excretion in the gut, taurine is thought to be converted to inorganic sulfate by the intestinal microbiota, which is then absorbed [72]. In human cells, it is unlikely that these reactions can take place and since taurine is also not metabolized further, taurine is often considered chemically and biologically inert [46,72]. As the kidneys are the primary regulators of taurine homeostasis and taurine is not metabolized further, a higher 24 h urinary excretion may very well reflect a higher intake or absorption of taurine from the gut. Indeed, studies have shown that urinary excretion of taurine is very low when dietary taurine is restricted, as in a vegetarian diet. On the contrary, in diets with a higher taurine content, urinary taurine excretion increases [71]. Therefore, an increased taurine intake may potentially improve graft survival in renal transplant recipients.

Strengths of this study include the large sample size of this well-defined and specific patient group of RTR, the long follow-up and the presence of appropriate controls. In addition, extensive data collection of many demographical and laboratory parameters enabled adjustment for many potential confounders. However, several limitations of this study need to be addressed. In general, statistical significance in observational studies suggests, but does not confirm, biologic significance. Whether the significant relation between urinary taurine excretion and graft failure in RTR is a causal or an associative relation remains to be determined. Due to the observational design of this study we also were unable to measure oxidative stress in the kidney, which precluded us from determining whether urinary taurine excretion was associated with oxidative stress in the kidney. Similarly, the observational design of this study does not allow for further studies to elucidate the biological mechanisms underlying the association of urinary taurine excretion with graft failure. Furthermore, the observational design of the study precluded us to demonstrate in an experimental way that urinary taurine excretion is not influenced by renal function. In addition, our study population consisted predominantly of Caucasian individuals, which calls prudence to extrapolation of our results to populations of other ethnicities. Lastly, the Dutch Food Composition Table did not include information on taurine content, which prevented us from comparing FFQ-based taurine intake to urinary excretion of taurine.

## 5. Conclusions

Higher urinary taurine excretion is associated with improved graft survival in renal transplant recipients. As taurine synthesis in humans is very limited, 24 h urinary taurine excretion largely reflects daily taurine intake. Therefore, an increased taurine intake may potentially increase graft survival in renal transplant recipients. However, further research is warranted to determine the mechanisms underlying this association and to investigate the potential of taurine supplementation in renal transplant recipients.

## Figures and Tables

**Table 1:** Baseline characteristics in 678 renal transplant recipients (RTR) and regression coefficients of the associations with log_2_ transformed urinary taurine excretion.

Variable	RTR Cohort (*n* = 678)	Range	Model 1		Model 2	
			Std. β	*P* value	Std. β	*P* value
**Taurine**						
Urinary taurine excretion, μmol/24 h	533 (210–946)	9–3637				
Urinary taurine concentration, μmol/L	216 (87–415)	3–1201	0.962	<0.001	0.953	<0.001
Urinary taurine/creatinine ratio, μmol/mmol	46 (20–80)	1–429	0.969	<0.001	0.993	<0.001
**Demographics**						
Age, years	53 ± 13	18–80	−0.007	0.86	0.019	0.58
Sex, n (% male)	390 (58)		−0.374	<0.001	−0.369	<0.001
Smokers, n (%)						
Never	265 (42)		0.083	0.04	0.113	0.002
Past	287 (45)		−0.071	0.07	−0.087	0.02
Current	82 (13)		−0.016	0.68	−0.038	0.29
Alcohol intake ^a^						
No alcohol	23 (4)		−0.037	0.36	−0.004	0.91
Low intake	400 (65)		0.001	0.98	0.029	0.42
High intake	197 (32)		0.014	0.73	−0.029	0.44
**Body composition**						
Weight, kg	80 ± 17	35–164	0.121	0.002	0.008	0.82
Height, cm	174 ± 10	127–197	0.266	<0.001	0.053	0.26
BMI, kg/m^2^	26.6 ± 4.8	15.7–45.0	−0.023	0.55	−0.005	0.88
BSA, m^2^	1.94 ± 0.22	1.09–2.83	0.196	<0.001	0.025	0.54
**Primary renal disease, n (%)**						
Primary glomerulosclerosis	194 (29)		−0.020	0.61	−0.051	0.14
Glomerulonephritis	49 (7)		0.009	0.82	0.012	0.73
Tubulointerstitial nephritis	83 (12)		−0.023	0.55	−0.014	0.70
Polycystic kidney disease	139 (21)		0.035	0.36	0.081	0.02
Hypo- or dysplasia	28 (4)		0.015	0.70	0.009	0.81
Renovascular disease	38 (6)		−0.020	0.61	−0.039	0.26
Diabetes	34 (5)		−0.002	0.96	0.004	0.92
**Cardiovascular history, n (%)**						
Coronary intervention	68 (10)		−0.005	0.90	−0.028	0.44
Myocardial infarction	34 (5)		0.036	0.35	0.031	0.38
CVA and/or TIA	41 (6)		−0.038	0.33	−0.012	0.73
**Cardiovascular**						
Systolic blood pressure, mmHg	136 ± 17	88–200	<0.001	1.00	−0.020	0.57
Diastolic blood pressure, mmHg	83 ± 11	50–125	0.043	0.27	−0.016	0.65
Mean arterial pressure, mmHg	107 ± 15	63–167	−0.002	0.95	−0.025	0.48
Pulse pressure, mmHg	54 ± 13	20–114	−0.037	0.34	−0.014	0.70
Heart rate, bpm	68 ± 12	41–122	−0.001	0.98	0.047	0.20
Hypertension, n (%)	275 (41)		0.037	0.33	0.002	0.95
Antihypertensive drugs, n (%)	595 (88)		−0.082	0.03	−0.062	0.09
Nt-proBNP, ng/L	247 (105–598)	1–110,000	−0.196	<0.001	−0.029	0.55
**Lipids**						
Total cholesterol, mmol/L	5.1 ± 1.1	2.3–9.7	−0.174	<0.001	−0.120	0.001
HDL cholesterol, mmol/L	1.4 ± 0.5	0.4–3.5	−0.112	0.004	−0.058	0.12
LDL cholesterol, mmol/L	3.0 ± 0.9	0.7–6.6	−0.094	0.02	−0.062	0.08
Triglycerides, mmol/L	1.7 (1.2–2.3)	0.3–8.5	−0.168	<0.001	−0.118	0.001
Statins, n (%)	360 (53)		−0.080	0.04	−0.063	0.08
**Glucose homeostasis**						
Glucose, mmol/L	5.3 (4.8–6.1)	2.1–21.7	0.002	0.97	−0.033	0.34
HbA1c, %	5.8 (5.5–6.2)	4.5–11.8	0.003	0.95	−0.019	0.61
Diabetes, n (%)	162 (24)		−0.059	0.12	−0.044	0.21
Antidiabetic drugs, n (%)	107 (16)		−0.093	0.02	−0.079	0.03
**Transplantation-related**						
Dialysis vintage, months	27 (10–52)	0–226	0.009	0.84	0.037	0.34
Time since transplantation, years	5.3 (1.8–11.5)	0.2–39	−0.169	<0.001	−0.180	<0.001
Deceased donor, n (%)	446 (66)		0.055	0.15	0.038	0.30
Cold ischemia time (hours)	15 (3–21)	0–40	−0.049	0.21	−0.028	0.44
Warm ischemia time (minutes)	40 (35–50)	10–128	0.035	0.37	0.024	0.49
Transplantations up to baseline, n (%)			−0.051	0.19	−0.018	0.62
1 transplantation	612 (90)					
≥2 transplantations	66 (10)					
Calcineurin inhibitors, n (%)	381 (56)		0.021	0.59	0.082	0.02
Proliferation inhibitor, n (%)	567 (84)		0.095	0.01	0.054	0.13
Prednisolone dosage, n (%)			0.135	<0.001	0.064	0.07
≤7.5 mg/24 h	275 (41)					
>7.5 mg/24 h	403 (59)					
HLA antibodies, n (%)						
HLA-I	106 (16)		−0.110	0.004	−0.026	0.46
HLA-II	114 (17)		−0.133	0.001	−0.067	0.06
**Renal function**						
Serum creatinine, μmol/L	124 (99–160)	50–591	−0.129	<0.001	−0.079	0.40
eGFR, ml/min/1.73m^2 b^	45 ± 19	7–107	0.252	<0.001	0.244	<0.001
Creatinine clearance, ml/min	66 ± 27	12–186	0.361	<0.001	0.314	<0.001
Proteinuria, n (%)	152 (22)		−0.107	0.005	−0.086	0.02
**Oxidative stress and inflammation**						
Free sulfhydryl groups (μmol/L)	132 ± 49	10–387	0.249	<0.001	0.126	0.001
hsCRP, mg/L	1.6 (0.7–4.5)	0.1–114	−0.037	0.35	0.006	0.88
**Urinary excretion**						
Sodium, mmol/24 h	158 ± 62	24–374	0.288	<0.001	0.179	<0.001
Chloride, mmol/24 h	138 (108–181]	28–391	0.268	<0.001	0.151	<0.001
Sulfate, mmol/24 h	17.6 ± 6.4	1.8–62.4	0.324	<0.001	0.199	<0.001
Thiosulfate, mmol/24 h	7 (4–12]	0–108	0.285	<0.001	0.170	<0.001
Creatinine, mmol/24 h	11.7 ± 3.4	2.9–23.3	0.397	<0.001	0.265	<0.001
**Dietary intake ^c^**						
Energy intake, kcal/24 h	2172 ± 619	658–4871	0.126	0.002	−0.038	0.34
Total protein intake, g/24 h	82 ± 12	26–144	0.190	<0.001	0.060	0.14
Animal protein intake, g/24 h	51 ± 13	18–110	0.133	0.001	0.085	0.03
Methionine intake, mg/24 h	1871 ± 327	687–3510	0.176	<0.001	0.077	0.05
Cysteine intake, mg/24 h	1187 ± 172	436–2296	0.238	<0.001	0.072	0.08
Meat intake, g/24 h	96 ± 38	0–275	0.223	<0.001	0.151	<0.001
Fish intake, g/24 h	11 (4–20)	0–134	0.104	0.01	0.096	0.008
Milk intake, g/24 h	117 ± 83	0–521	−0.005	0.90	0.016	0.66
Plant protein intake, g/24 h	31 ± 6	7–72	0.110	0.006	−0.077	0.06
Vegetable intake, g/24 h	93 ± 57	0–412	0.021	0.61	0.034	0.35
Fruit intake, g/24 h	152 ± 114	0–621	−0.117	0.004	−0.072	0.05
Total fat intake, g/24 h	88 ± 16	21–256	0.212	<0.001	−0.005	0.91
Saturated fat intake, g/24 h	31 ± 7	7–97	0.119	0.003	−0.021	0.59
Monounsaturated fat intake, g/24 h	30 ± 7	7–97	0.237	<0.001	0.016	0.72
Polyunsaturated fat intake, g/24 h	19 ± 6	4–74	0.151	<0.001	−0.026	0.52
Total carbohydrate intake, g/24 h	249 ± 63	16–537	0.186	<0.001	0.037	0.35
Mono and disaccharides intake, g/24 h	121 ± 34	9–301	0.106	0.008	−0.006	0.87
Polysaccharides intake, g/24 h	127 ± 28	8–297	0.190	<0.001	−0.009	0.83

Model 1: Crude, Model 2: Crude with adjustment for age, sex and eGFR. Urinary taurine excretion was log_2_-transformed for analysis. Regression coefficients are given as standardized beta values (Std. β), the latter referring to the number of standard deviations a dependent variable changes per standard deviation increase of the independent variable, thereby allowing for comparison of the strength of the associations of different variables. ^a^ Low alcohol intake was defined as 0–5 g/24 h in females, and 0–10 g/24 h in males. High alcohol intake was defined as >5 g–24 h in females and >10 g–24 h in males). ^b^ For eGFR, model 2 was adjusted only for age and sex. ^c^ Dietary intake was adjusted for energy intake through the residual method. Dietary intake range was shown for the unadjusted values. Abbreviations: BMI: body mass index; BSA: body surface area; CVA: cerebrovascular accident; TIA: transient ischemic attack; HDL: high-density lipoprotein; LDL: low-density lipoprotein; HLA: human leukocyte antigen; eGFR: estimated glomerular filtration rate; hsCRP: high-sensitivity C-reactive protein.

**Table 2:** Association of urinary taurine excretion, urinary taurine concentration and urinary taurine creatinine ratio with death-censored graft failure.

Model	Urinary Taurine Excretion		Urinary Taurine Concentration		Urinary Taurine/Creatinine Ratio	
	HR (95% CI)	*P* Value	HR (95% CI)	*P* Value	HR (95% CI)	*P* Value
Model 1	0.74 (0.67–0.82)	<0.001	0.75 (0.67–0.84)	<0.001	0.74 (0.66–0.83)	<0.001
Model 2	0.83 (0.74–0.93)	0.002	0.84 (0.74–0.95)	0.007	0.85 (0.75–0.96)	0.010
Model 3	0.82 (0.71–0.95)	0.01	0.83 (0.71–0.97)	0.02	0.84 (0.72–0.98)	0.03
Model 4	0.84 (0.74–0.95)	0.004	0.86 (0.76–0.98)	0.02	0.86 (0.76–0.98)	0.02
Model 5	0.84 (0.74–0.95)	0.006	0.84 (0.74–0.96)	0.01	0.84 (0.74–0.96)	0.01

Model 1. Log_2_-transformed urinary taurine excretion, urinary concentration or urinary taurine/creatinine ratio (crude). Model 2. Model 1 + basic confounders (age, sex, weight, height, eGFR, proteinuria). Model 3. Model 2 + cardiovascular risk factors (total cholesterol, HDL cholesterol, triglycerides, systolic blood pressure, antihypertensive treatment, smoking (current, ex, or never), diabetes, medical history of coronary intervention, medical history of myocardial infarction, medical history of CVA and/or TIA) and alcohol intake. Model 4. Model 2 + transplantation related factors (donor type, total dialysis time, time between transplantation and baseline, cold ischemia time, calcineurin inhibitor usage, proliferation inhibitor usage, and the number of transplantations up to baseline). Model 5. Model 2 + polycystic kidney disease, urinary excretion of sodium, chloride, sulfate, thiosulfate and creatinine. Abbreviations: HR: hazard ratio; CI: confidence interval; eGFR: estimated glomerular filtration rate; HDL: high-density lipoprotein; CVA: cerebrovascular accident; TIA: transient ischemic attack.

## Supplementary Materials

The following are available online at https://www.mdpi.com/2072-6643/11/9/2212/s1, Table S1. Characteristics of 678 RTR and 275 controls at the day of their visit to the outpatient clinic. Healthy kidney donors are used as controls. Table S2. Association of urinary taurine excretion, urinary taurine concentration and urinary taurine creatinine ratio with death-censored graft failure, using creatinine clearance instead of eGFR to adjust for renal function.
