# Supplementary material for: Urinary Taurine Excretion and Risk of Late Graft Failure in Renal Transplant Recipients
Source: Nutrients. 2019 Sep 13;11(9):2212. doi: 10.3390/nu11092212 (PMC6770760; doi:10.3390/nu11092212)
Supplement: Supplementary file 1 [file nutrients-11-02212-s001.pdf]

Supplementary data

Table S1. Characteristics of 678 RTR and 275 controls at the day of their visit to the outpatient clinic. Healthy kidney donors are used as controls.

	RTR (n=678)	Controls (n=275)	P value
**Taurine**			
Urinary taurine excretion, μmol/24h	533 (210-946)	477 (253-943)	0.92
Urinary taurine concentration, μmol/L	216 (87-415)	199 (100-394)	0.85
Urinary taurine / creatinine ratio, μmol/mmol	46 (20-80)	41 (21-66)	0.21
**Demographics**			
Age, years	53 ± 13	54 ± 11	0.52
Sex, n (% male)	390 (58)	129 (47)	0.002
Smokers, n (%)			
Never	265 (42)	111 (53)	<0.001
Past	287 (45)	49 (24)	
Current smoker	82 (13)	48 (23)	
**Body composition**			
Weight, kg	80 ± 17	73 ± 14	0.32
Height, cm	174 ± 10	175 ± 10	0.12
BMI, kg/m^2	26.6 ± 4.8	26.0 ± 3.5	0.02
BSA, m^2	1.94 ± 0.22	1.94 ± 0.20	0.85
**Medication usage**			
Antihypertensive drugs, n (%)	595 (88)	8 (5)	<0.001
Statins, n (%)	360 (53)	31 (17)	<0.001
**Renal function parameters**			
Serum creatinine, μmol/L	124 (99-160)	73 (65-82)	<0.001
eGFR, ml/min/1.73m^2	45 ± 19	92 ± 16	<0.001
Creatinine clearance, ml/min	66 ± 27	126 ± 39	<0.001
Proteinuria, n (%)	152 (22)	1 (0.4)	<0.001
**Dietary intakes ***			
Energy intake, kcal/24h	2172 ± 619	2294 ± 730	0.02
Total protein intake, g/24h	82 ± 12	85 ± 12	0.007
Animal protein intake, g/24h	51 ± 13	51 ± 12	0.98
Plant protein intake, g/24h	31 ± 6	33 ± 8	<0.001
Methionine intake, mg/24h	1871 ± 327	1920 ± 322	0.07
Cysteine intake, mg/24h	1187 ± 172	1238 ± 190	0.001
Total fat intake, g/24h	88 ± 16	94 ± 20	<0.001
Total carbohydrate intake, g/24h	249 ± 63	258 ± 49	0.03

P value for difference was tested by independent t test, Mann Whitney U or chi-squared test
* Dietary intake was adjusted for energy intake through the residual method. Dietary intake was available in 641 RTR and 183 controls.

Table S2. Association of urinary taurine excretion, urinary taurine concentration and urinary taurine creatinine ratio with death-censored graft failure, using creatinine clearance instead of eGFR to adjust for renal function.

	Urinary taurine excretion		Urinary taurine concentration		Urinary taurine/creatinine ratio	
	HR [95% CI]	P value	HR [95% CI]	P value	HR [95% CI]	P value
Model 1	0.74 [0.67-0.82]	<0.001	0.75 [0.67-0.84]	<0.001	0.74 [0.66-0.83]	<0.001
Model 2	0.84 [0.74-0.95]	0.005	0.84 [0.74-0.96]	0.007	0.83 [0.73-0.93]	0.002
Model 3	0.85 [0.73-0.98]	0.03	0.84 [0.72-0.98]	0.02	0.82 [0.70-0.95]	0.009
Model 4	0.85 [0.75-0.97]	0.012	0.86 [0.76-0.98]	0.02	0.84 [0.74-0.95]	0.007
Model 5	0.82 [0.72-0.93]	0.002	0.83 [0.73-0.94]	0.003	0.82 [0.73-0.94]	0.003

Model 1	Log_2-transformed urinary taurine excretion, urinary concentration or urinary taurine/creatinine ratio (crude).
Model 2	Model 1 + basic confounders (age, sex, weight, height, creatinine clearance, proteinuria).
Model 3	Model 2 + cardiovascular risk factors (total cholesterol, HDL cholesterol, triglycerides, systolic blood pressure, antihypertensive treatment, smoking (current, ex, or never), diabetes, medical history of coronary intervention, medical history of myocardial infarction, medical history of CVA and/or TIA) and alcohol intake.

Model 4	Model 2 + transplantation related factors (donor type, total dialysis time, time between transplantation and baseline, cold ischemia time, CNI usage, proliferation inhibitor usage, and the number of transplantations up to baseline).
Model 5	Model 2 + polycystic kidney disease, urinary excretion of sodium, chloride, sulfate, thiosulfate and creatinine,

Abbreviations: eGFR: estimated glomerular filtration rate; HDL: high-density lipoprotein; CVA: cerebrovascular accident; TIA: transient ischemic attack; CNI: calcineurin-inhibitor;
